# Healthcare Professionals’ Practice of HIV Post-Exposure Prophylaxis in Clinical Settings in Karachi, Pakistan

**DOI:** 10.3390/healthcare10020277

**Published:** 2022-01-30

**Authors:** Sadia Shakeel, Wajiha Iffat, Saima Naseem, Shagufta Nesar, Hina Rehman, Muhammad Yaqoob, Anees Ur Rehman, Ibrahim Barrak, Shazia Jamshed, Márió Gajdács

**Affiliations:** 1 Department of Pharmacy Practice, Faculty of Pharmaceutical Sciences, Dow College of Pharmacy, Dow University of Health Sciences, Karachi 74200, Pakistan; sadia.shakeel@duhs.edu.pk; 2 Department of Pharmaceutics, Faculty of Pharmaceutical Sciences, Dow College of Pharmacy, Dow University of Health Sciences, Karachi 74200, Pakistan; wajiha.iffat@duhs.edu.pk; 3 Dow International Medical College, Dow University of Health Sciences, Karachi 74200, Pakistan; saima.naseem@duhs.edu.pk; 4 Jinnah College of Pharmacy, Sohail University, Karachi 74200, Pakistan; shaguftausmani@sohailuniversity.edu.pk; 5 Department of Pharmacy Practice, Institute of Pharmaceutical Sciences, Jinnah Sindh Medical University, Karachi 74200, Pakistan; hina.rehman@jsmu.edu.pk; 6 Dow Institute of Nursing and Midwifery, Dow University of Health Sciences, Karachi 74200, Pakistan; muhamad.yaqub@duhs.edu.pk; 7 Department of Pharmacy Practice, Faculty of Pharmacy, Bahauddin Zakariya University, Multan 60800, Pakistan; aneesurrehmanr90@gmail.com; 8 Department of Periodontology, Faculty of Dentistry, University of Szeged, 6720 Szeged, Hungary; barrakibrahim@gmail.com; 9 Department of Clinical Pharmacy and Practice, Faculty of Pharmacy, Universiti Sultan Zainal Abidin, UniSZA, Kuala Terengganu 20400, Malaysia; shaziajamshed@unisza.edu.my; 10 Department of Oral Biology and Experimental Dental Research, Faculty of Dentistry, University of Szeged, 6720 Szeged, Hungary

**Keywords:** healthcare professionals, human immunodeficiency virus, HIV, post-exposure prophylaxis, occupational exposure, Pakistan

## Abstract

The human immunodeficiency virus (HIV) is an important public health concern that has become more prevalent in Pakistan in recent decades. Healthcare professionals (HCPs) are frequently exposed to many HIV-infected patients; as a result, they are more vulnerable to HIV infection due to occupational exposure. Hence, the current study was executed to evaluate HCPs’ knowledge, attitude and practice in terms of post-exposure prophylaxis (PEP) for HIV. This cross-sectional study was carried out in several clinical and laboratory settings of Karachi and the HCPs involved in treating patients were surveyed using a structured questionnaire. The Shapiro–Wilk test was performed to establish the normality of the variables. Pearson correlation was employed to identify the relationship between the independent variables considering *p*-values < 0.05 as statistically significant. A total of 578 filled forms were incorporated in the study with a response rate of 72.2%. Physicians and medical students (OR = 1.68; 95% CI = 1.16–2.24; *p* = 0.001) belonging to private work settings (OR = 1.84; 95% CI = 1.33–2.35; *p* < 0.003) indicated better knowledge. The majority, 407 (70.4%), of the respondents reported having been exposed to risky occupational circumstances during their professional life; however, 65.7% took PEP for HIV after exposure and only 56.8% completed the entire course. A statistically significant association was observed between experience (*p* = 0.004, CI = 0.14–0.72), job category (*p* = 0.0001, CI = 0.16–0.62) and frequency of exposure (*p* = 0.003, CI = 0.42–11.31) and reporting of occupational exposure. More than half (53.8%) of respondents stated that their institute has a policy for the management of HIV exposures; however, their response was significantly associated with their organization (*p* = 0.004). The current study shows adequate knowledge revealing a positive attitude among respondents; however, there was a gap between the knowledge and its practical application. Even though many of the HCPs had experienced risky HIV exposure, a lack of reporting was noted in the study.

## 1. Introduction

Infections caused by the Human Immunodeficiency Virus (HIV) are a progressively rising risk to global public health, threatening the lives of millions of individuals across the world. Pakistan is among those nations in the World Health Organization (WHO) Eastern Mediterranean Region where the incidence of HIV infections and its death toll has shown a progressively increasing trend every year since 1987 [1]. The overall number of cases of HIV infections in Pakistan has been growing at a distressing rate; particularly, from 2005 to 2015, the integer of reported cases in Pakistan ballooned from 8360 to 45,990, the top global average rise (17.6%) in recorded history [2]. In the past two decades, up to eight outbreaks of HIV have been reported in Pakistan, with a single district—Larkana—being the most affected [2]. In 2018 alone, 21,000 new cases of people living with HIV (PLHIV) were documented. In April 2019, local media reports in Pakistan informed the Sindh AIDS Control Program (SACP) about 14 children aged 10 years with new HIV diagnoses in Ratodero, a rural sub district of Larkana District in Sindh province [3]. By 18 May 2019, health officials had discovered 571 (3.4 percent) new instances of HIV infection among 16,856 people tested, 463 (81 percent) of which were in children and adolescents aged 15 years, including 355 (62 percent) in children under 5 years [4]. Because of insufficient information flow and scarce diagnostic facilities, estimates of PLHIV are substantially higher than the actual number of recorded cases [5]. The preliminary investigations (including patient interviews, site visits to clinics, hospitals and blood banks and a review of surveillance data) identified unsafe injection practices at health care facilities, unsafe practices at blood banks, inadequate infection control measures and improper medical waste management as the possible risk factors. Pakistan’s National AIDS Control Program (NACP) offers preventative, diagnostic and therapeutic services through the country’s hospital-based infrastructure. The NACP has established 19 HIV treatment facilities, all of which are located in hospitals, in partnership with their provincial equivalents. The SACP advised that infection prevention and control, as well as blood safety, need to be improved by training health care personnel in terms of safe injection techniques, forming task groups with key stakeholders and implementing policy reforms and laws [4]. In a society with a low literacy rate, where many health views are driven by myths and fallacies and with a poorly underutilized public health system, illnesses such as HIV take on a much larger magnitude due to financial, social and political obstacles [5].

Healthcare professionals (HCPs) are at an elevated risk of occupational exposure to HIV-infected patients. As they have a forefront role in patientcare, they are in direct interaction with patients and their bodily fluids and blood and they are at a greater possibility of occupational exposure to HIV/AIDS and other infections. The WHO indicated that over 3 million percutaneous occupational injuries take place every year among HCPs all over the world [5]. The chances of contracting HIV by percutaneous contact with HIV-infected blood and mucous membrane contact were estimated to be around 0.3% and 0.09%, respectively [6]. The likelihood of HIV transmission after occupational exposure hinges on various factors, for instance, the viral titer in the blood of the source individual, the nature of the injury, the amount of body fluid or blood transmitted to the HCP all through the exposure and the immune status of the exposed HCP [7]. Post-exposure prophylaxis (PEP) includes the administration of a course of antiretroviral therapy (ART) after procedures having a higher possibility of HIV exposure [8]. The appropriate use of ART as a prophylactic measure decreases the possibility of getting HIV infection by 81% following exposure [9]. PEP against HIV infection generally comprises first aid measures after exposure, counseling, risk assessment and laboratory examinations, accompanied by consent from the source and exposed people, followed by 28 days of ART and monitoring [10,11].

The most effective safety measure to prevent contamination with body fluids and blood include precautionary and safety measures, safe-needle procedures, barriers and other preventive strategies [12]. Global safety measures for all HCPs include using gloves and other protective barriers while at risk for exposure to blood or other body fluids, non-intact skin or mucous membranes of the patients, as well as avoidance of injuries from needles, blades, or other sharp instruments [13,14]. Being a developing country, deprived of essential healthcare resources and the accessibility of preventive measures needed for the protection of HCPs, prevention of the transmission of such exposures in Pakistan is still insufficient, driving the HCPs among the high-risk groups occupationally exposed to HIV. An understanding of HCPs’ skills and performance may be crucial to evaluate and curtail the risk of occupational exposure to HIV amongst these individuals [15]. No reliable database nor statistics exists that depicts the prevalence of occupational mishaps, the practice of reporting such exposures and the use of PEP against HIV amongst HCPs in Pakistan. Hence, the current study was executed to evaluate HCPs’ knowledge, attitude and practice (KAP) in terms of PEP for HIV in the metropolitan city of Karachi, Pakistan.

## 2. Materials and Methods

### 2.1. Study Design and Population

This cross-sectional study was carried out from January 2020 to July 2020. The responses were collected from four public and seven private sector hospitals/clinics, thirteen laboratory and diagnostic centers and two health science universities in Karachi, Pakistan, by providing the survey forms to the respondents. The particular sites for recruiting the respondents were selected on the basis of them permitting us to conduct the research study; we ensured that the HCPs working in selected organizations would provide the information necessary to address the research questions of the current study. Ethical approval was acquired from the Institutional Review Board of Darul Sehat Hospital, Karachi, with the protocol number DSH/IRB/2020/0023. Written consent was obtained from all the respondents and the objectives of the study were clarified to them before participation. The study was carried out using the convenience and snowball sampling method. The respondents were approached through email, different social media platforms or personal contacts and were invited to complete the survey form.

### 2.2. Inclusion and Exclusion Criteria

HCPs (physicians, nurses, medical students and lab staff, including biochemists, microbiologists and lab technicians) who were legitimately involved in the care of patients and willing to participate were selected as the targeted population for the study. The HCPs not directly involved in patientcare, or those who declined to take part were excluded.

### 2.3. Sampling Technique

The Raosoft sample size calculator was used for calculating the required sample size for this study [16]. The response rate in the current study was estimated to be 50%. The standard deviation (SD) was set at 1.96 for a 95% confidence interval (CI). The margin of error was set at ±5%. Based on these parameters, the recommended sample size was set at *n* = 385. The design effect was set at 1.0 because of the homogeneity of the respondents in the participating institutions. Taking into account the expected response rate based on the pilot study—which was 95%—the sample size was increased and a total of *n* = 800 questionnaires were distributed among the HCPs.

### 2.4. Study Tool

The researchers surveyed respondents by using a questionnaire derived from a review of previous studies [6,7,12,13] to assess occupational exposures to HIV-infected patients and their reporting practices, as well as their KAP in terms of seeking PEP for HIV. The questionnaire was pre-tested on a smaller percentage of the respondents (*n* = 30) to evaluate the precision and transparency of questionnaire items and to verify content validity (face validity). The Cronbach’s alpha was calculated to determine the instrument’s internal consistency; the Cronbach’s alpha value was found to be 0.831, which was sufficient to meet the current study’s aims. Following the pilot testing, a minor modification to the questionnaire was required for improved clarity and understandability. The HCPs who participated in the pilot study were not included in the final study.

Besides the demographic information, the survey form included 23 closed-ended questions to evaluate HCPs’ KAP in terms of managing and reporting the occupational HIV exposures and their utilization of PEP. Twelve questions were prepared to observe the practices followed for the management of occupational exposures in the institutes of the respondents. For correct and incorrect responses, score of 1 or 0 were assigned, accordingly, for each of the 10 knowledge questions. The overall knowledge score, considered as the sum of all specific scores, ranged from 0 to 10 points, with greater scores representing better knowledge concerning PEP. Using cutoff points at 50% and 75% of the total score, the respondents’ knowledge was categorized as poor (0–5), average (6–7), or good (8–10).

### 2.5. Statistical Analysis

The data entry and analyses of the responses collected were performed using the Statistical Package for Social Sciences v.20.0 (SPSS; Chicago, IL, USA). The demographic data of the respondents were illustrated as percentages and frequencies. The Shapiro–Wilk test was performed to establish the normality of the variables. Pearson correlation coefficients were employed to identify the relationship between the independent variables and the responses considering *p*-values < 0.05 as statistically significant. To evaluate the association among knowledge and attitudes of HCPs, the Spearman correlation coefficient (*p* < 0.05) was applied.

## 3. Results

In the present study, the researchers provided 800 survey questionnaires to the responders; *n* = 592 surveys were returned. Fourteen (*n* = 14) questionnaires were excluded from the study because the attached consent form was not filled. Thus, a total of *n* = 578 completed questionnaires were incorporated in the study; hence, the overall response rate was 72.2%. Among respondents, females were in the majority (63.6%; *n* = 368) (Table 1). The respondents included physicians (43%; *n* = 249), nurses (2.7%; *n* = 16), medical students in their clinical module (30.2%; *n* = 175) and laboratory staff (including biochemists, microbiologists and laboratory technicians) (23.8%; *n* = 138). More than half (58.4%; *n* = 338) of the respondents worked in public sector’s clinical/laboratory settings. The mean age of the respondents was 34.2 ± 11.3 years and (43.7%; *n* = 253) respondents reported having work experience of less than 5 years. Most of the respondents, including clinical pathologists, consultant physicians and lab staff, reported working in laboratories (55.0%; *n* = 318), in addition to academic institutions (28.0%; *n* = 162), hospitals (7.9%; *n* = 46) and clinics/private practices 23 (3.9%; *n* = 23). Respondents’ major source of information regarding PEP was continuous medical education (CME)/training (35.9%; *n* = 208) followed by peers/friends 173 (29.9%).

The median (interquartile range, IQR) knowledge score based on the HCPs responses was 7.9 (6.5–9.3), signifying an overall 74.1% appropriate knowledge rate in terms of PEP for HIV. However, their knowledge was significantly associated with their experience (*p* = 0.006) and the nature of their job/occupation (*p* = 0.002). Physicians and medical students (OR = 1.68; 95% CI = 1.16–2.24; *p* = 0.001) belonging to private work settings (OR = 1.84; 95% CI = 1.33–2.35; *p* < 0.003) showed better PEP-related knowledge. In comparison, laboratory technicians rendering their services in the public sector were found to be less conversant (OR = 0.60; 95% CI = 0.45–0.75; *p* = 0.005). More than 80% stated that they had attended training regarding PEP (Table 2). There was no significant association with age (*p* = 0.96), gender (*p* = 0.72), nor integer of exposures experienced by the respondents (*p* = 0.82) and PEP knowledge scores. The major causes stated as a reason for exposure were: high workload (44.2%; *n* = 256), shortage of protective barriers (33.2%; *n* = 192) and deficiency of knowledge on standard precautions (17.6%; *n* = 102). Around 89% of HCPs thought that there should be PEP guidelines present in the working areas (Table 3).

**Figure 1:**
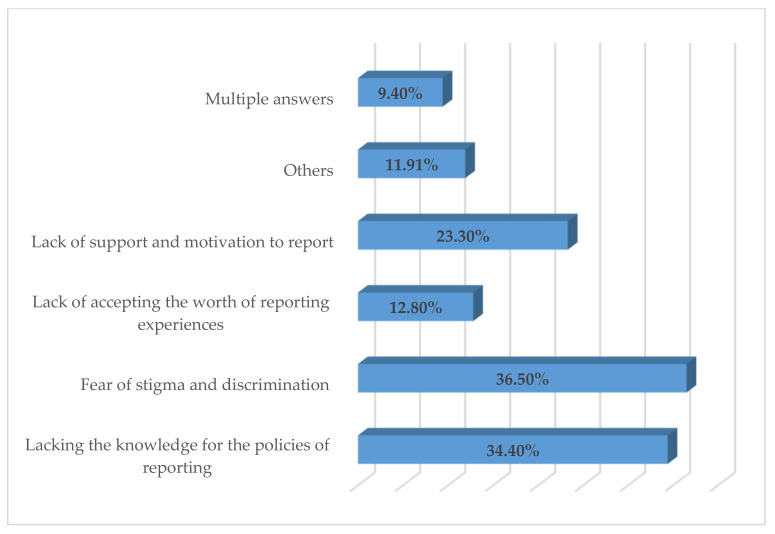
Respondents’ barriers for reporting exposure.

Concerning exposure to the risk of acquiring HIV, *n* = 407 (70.4%) of the respondents reported having been exposed to risky circumstances during their work (Table 4). However, *n* = 380 (65.7%) took PEP after exposure. A majority of respondents (60.8%; *n* = 352), considered the type of exposure, the bodily fluid, the patient’s HIV status and the exposed person’s susceptibility as the factors to be considerable for follow-up after occupational exposure. Among the *n* = 407 respondents who had been exposed to risky circumstances, only *n* = 356 (61.5%) reported the exposures verbally to the concerned authority in their institutes; however, none of these exposures were reported as suggested by WHO recommendations. The 38.5% of exposed respondents who did not notify their experiences provided similar justifications as given by the remaining respondents as reasons that may hinder one from reporting exposure. The major reasons for not reporting the occupational exposures by the respondents were: lacking the knowledge of policies for reporting (34.4%; *n* = 199), fear of stigma and discrimination (36.5%; *n* = 211), lack of support and motivation to report (23.3%; *n* = 135) and lack of accepting the worth of reporting experiences (12.8%; *n* = 74) (Figure 1). A statistically significant association existed between experience (*p* = 0.004, CI = 0.14–0.72), job category (*p* = 0.0001, CI = 0.16–0.62) and frequency of exposure (*p* = 0.003, CI = 0.42–11.31) and tendency of reporting occupational exposure (Table 5).

Table 6 illustrates the respondents’ opinions about the management of occupational exposures in the healthcare institutions they worked in. More than half (53.8%; *n* = 311) stated that their institutions had a policy for the management of HIV exposures; however, their response was significantly associated with the type of organization they were employed by (*p* = 0.004). More than half (60.8%; *n* = 352) responded that their institute provided training to all staff on the actions needed to be taken in response to occupational exposures; here, the response was also significantly associated with organization type (*p* = 0.001). A significant difference was observed in the knowledge score (9.34 vs. 7.41, *p* = 0.004) and practices (6.72 vs. 4.13, *p* = 0.001) of HCPs having greater than 10 years of experience with those having less than 10 years of experience. The Spearman correlation test revealed a weak, but significant positive association between the knowledge and attitude of HCPs towards PEP (r = 0.214, *p* < 0.005).

## 4. Discussion

The current study depicts the aspects of KAP in terms of using PEP for HIV in a developing country with low socio-economic standards. Pakistan has been characterized by inadequate measures to combat HIV/AIDS, as the UN has listed it as one of 11 nations with the highest incidence of the illness—13%—in its latest report [4]. It is the highest HIV prevalence ratio the country has seen in the previous decade, prompting worries among global health associates. According to the UNAIDS 2019 report, the world is on course to stop the AIDS epidemic by 2030, but Pakistan is one of the nations where the ratio of new AIDS cases has increased dramatically [3,4]. Thus, studies elaborating on the knowledge, attitude and practices of HCPs—being at high-risk—towards HIV post-exposure measures are warranted [17,18].

In the current study, the convenience and snowball sampling methods were used for survey distribution, as convenience sampling provides a swift turn-around of responses. The overall response rate was 72.2%, in contrast with the 100% response rate of a study conducted in an Ethiopian hospital [19]. The lower response rate might be due to the longer survey questionnaire, which may have had a major impact on the survey response. The study focused on the perspectives and practices of HCPs who were legitimately involved in the care of patients. However, the number of participating nurses was low (2.7%), considering that this category of professionals spends more time in contact with patients. It might be because low survey response rates have long been an issue in research that encompasses nursing and midwifery. The majority of respondents had adequate knowledge and expressed an optimistic attitude towards the importance of timely use of PEP; however, their knowledge was significantly associated with their experience (*p* = 0.006) and the nature of the job (*p* = 0.002). Another study reported a similar significant association between PEP knowledge and the level of respondents’ education (*p* < 0.001) [20]. It was observed that physicians and medical students had a better understanding of PEP than laboratory workers and nurses. This might be because physicians go through a lengthy period of education and training, together with frequent and compulsory CME and workshops planned for physicians. The proportion of HCPs with low knowledge in the current study was 25.9%, which is higher than the results of the previous study conducted in South Africa [21]. Kabotho et al. reported poor knowledge of PEP, revealing that 58% of respondents showed inappropriate knowledge of PEP and only 27% of HCPs were classified as having adequate knowledge [21]. Based on our results, the majority of respondents (93.5%) agreed that PEP is essential and 92.9% believed that PEP is critical in preventing further infection. This is comparable to another study, in which more than half (55.6%) of the participants showed an optimistic approach [22]; on the other hand, our results is conflicting with the results of the study conducted by Alawad et al., where less than half of the respondents revealed a progressive approach [23]. The majority of the respondents in the current survey had an affirmative approach toward HIV PEP. Perhaps, this is due to their adequate level of knowledge about PEP. In the current study, the respondents believed, regarding the nature of occupational expose to HIV-infected blood, that the highest possibility for the spread of infection is a percutaneous injury (43.1%), whereas 23.8% and 16.2% opined that skin contact with HIV-infected blood and a mucous membrane exposure, respectively, pose the greatest risk for infection transmission. One more study reported that the threat of transmission of HIV infection associated with percutaneous contact with infected blood is ~0.3% for every exposure [18].

When asking the respondents about the time of starting PEP for HIV, 57% of the respondents specified that PEP must be taken in an hour; however, a considerable portion of the respondents replied that the best time to take PEP is within 72 h after exposure, which is in line with the outcomes of another study, in which 58.5% respondents correctly noted the suggested time to take PEP and 41.5% correctly replied about the duration of complete PEP [21]. A similar investigation showed that 87.5% of respondents expressed precisely when to start PEP, which is a higher rate than our reported response rate [24]. A study conducted in India demonstrated that only 5.1% of the study participants took PEP; however, none of them finished the course, which is noteworthy when compared to the present investigation [25]. The disparity may be a result of the differentiation in awareness of respondents and the time variance between the conducted studies.

In the current study, the major causes stated as reason for exposure to HIV risk in the workplace were high workload (44.2%; *n* = 256), shortage of protective barriers (33.2%; *n* = 192) and lack of knowledge of standard precautions (17.6%; *n* = 102). Similar findings were reported by a similar Pakistani study demonstrating stress or being overburdened, followed by careless attitude as the most commonly identified reasons [26]. Regarding the exposure to the risk of acquiring HIV, 70.4% (*n* = 407) of HCPs reported having been exposed to occupationally risky circumstances. The percentage is higher than that illustrated in another study showing that half of the respondents had a minimum of one work-related exposure in recent times [17]. HCPs may be prone to infections because of their unsafe practices; for instance, insensible handling of soiled needles, reutilization of imperfectly sterilized needles, inappropriate dumping of hazardous leftovers and overpopulation of patient care areas are all highly inappropriate practices [7]. Nevertheless, such infections are mainly preventable by adopting stringent infection control procedures, for instance, the use of safety devices, correct approaches of medical waste dumping, immunization and swiftly managing exposures with the use of PEP [10]. However, HCPs sometimes fail to follow all-inclusive safety measures and have risky practices in healthcare settings for a variety of reasons. This is comparable with the outcomes of another study performed amongst medical students [27]. A paper reporting on results from Pakistan showed that the practices of nurses were safe in comparison to those of doctors; in addition, nurses were observed to be more vigilant in following protocols and guidelines during their clinical practice [28]. About 75% of the respondents in our study agreed that their institutes must initiate the trend of reporting and managing occupational exposures to ensure well-timed and appropriate responses; however, their response was significantly associated with the nature of their job (*p* = 0.0001). Among the respondents, 61.5% reported to have experienced exposure in their work as an HCP, which is in contrast with previous reports, where only 27.8% respondents reported to have experienced exposure [27]. Though a decent figure of respondents reported their risky occupational exposures, the HCPs’ reported practices were not as per the recommendations of the WHO [27]. This might be due to the lack of a proper pathway for reporting or the nonexistence of an appropriate procedure for reporting occupational mishaps amongst HCPs. A higher percentage of HCPs mentioned that the nonexistence of an appropriate reporting channel was the major cause for not reporting their exposure experiences. The shortage of information on exposure to HIV amongst HCPs in Pakistan supports these results.

The WHO suggests the timely use of PEP following occupational exposure associated with a HIV-infected patient [29]. In this study, only 30% of the respondents stated that their working organizations had developed guiding principles for the choice and usage of PEP ART for HIV occupational exposure and PEP was taken by approximately 65.7% of the respondents who reported exposure. This ratio for the uptake of PEP is greater than that reported in the study by Aschale et al., in which about 43.3% of study participants took PEP [30]. The major reason for the discontinuation of the drug in the current study was partly due to the assumption that the drug was not effective and due to the fear of side effects accompanying the medications administered as PEP, particularly for those used in HIV. This outcome is consistent with findings found in another study, which likewise noted a less pronounced use of PEP owed to the side effects of the medications administered [31]. Another study reported the fear of side effects as the major reason for not taking PEP [20]. The discontinuation of PEP can likewise be explained by the knowledge of the respondents of PEP, noted among HCPs. An alternate reason might be the sero-negativity of the source individual, which may have been subsequently verified. The HCPs’ cognizance concerning the negative results of the source individual may impact the use and non-adherence to PEP.

The strength and highlight of the current study are that it was conducted in the healthcare settings of the metropolitan city of Karachi. The study outcomes may support relevant health authorities in healthcare settings to further advance the knowledge of PEP against HIV, associated exposure risks and use of PEP for HCPs employed in relevant health facilities in the region. Nevertheless, the study has some limitations. Firstly, the nature of the study is cross-sectional, which is—by its nature—limited in establishing the causal pathways. Because the HCPs were examined over a specific time period in Karachi, the results may not be generalizable to all HCPs in Pakistan. Furthermore, the study’s findings are based on self-reported data and it might have been difficult for respondents to recall their specific exposure status. Thus, there is a probability and risk of over-reporting or underreporting associated with the results.

## 5. Conclusions

Our study reveals that, in the sample of Pakistani HCPs, adequate knowledge was reported regarding HIV PEP; however, there was a gap between the knowledge and its practical application (i.e., a “knowledge-practice” gap). Even though many of the HCPs had had potentially risky exposures to HIV, a lack of reporting of occupational exposure and less common use of PEP was noted in the study. Hence, training HCPs on infection control measures at work, in conjunction with a standard structure for adequate reporting of occupational exposures, is advised.

## Figures and Tables

**Table 1:** Characteristics of study population.

Characteristics	Frequency (*n*, %)
Gender	
Male	210 (36.3)
Female	368 (63.6)
Working Organization	
Private	240 (41.5)
Public sector	338 (58.4)
Job Category	
Physicians	249 (43.0)
Nurses	16 (2.7)
Medical students	175 (30.2)
Lab Staff	138 (23.8)
Work Experience (Years)	
Less than 5	253 (43.7)
Between 5 and 10	167 (28.8)
Between 10 and 15	64 (11.0)
Between 16 and 20	49 (8.4)
21 and above	45 (7.7)

**Table 2:** Respondents’ knowledge of PEP for HIV.

Respondents’ knowledge about PEP	Yes (*n*, %)
Definition of PEP	517 (89.4)
Training	468 (80.9)
Awareness of guidelines	413 (71.4)
PEP is essential	541 (93.5)
Importance of PEP for preventing infection	537 (92.9)
**When do you think PEP should be used?**	**Yes (*n*, %)**
When the source person is at a higher risk of contracting HIV	241 (41.6)
When an individual is found to be HIV-positive	352 (60.8)
When an individual’s HIV status is unknown	71 (12.2)
In the event of a needlestick injury at work	170 (29.4)
Multiple responses	213 (36.8)
**What is the maximum time to delay taking PEP?**	**Yes (*n*, %)**
12 h	71 (12.2)
24 h	60 (10.3)
48 h	53 (9.1)
72 h	394 (68.1)
**When is the best time to take PEP?**	**Yes (*n*, %)**
Within an hour of exposure	330 (57.0)
Within 6 h of exposure	69 (11.9)
Within 12 h of exposure	56 (9.6)
Within 72 h of exposure	123 (21.2)
**What is the effectiveness of PEP?**	**Yes (*n*, %)**
100%	89 (15.3)
80–100%	352 (60.8)
60–70%	53 (9.1)
30–50%	53 (9.1)
20–30%	31 (5.3)
**What is the time period during which PEP should be taken?**	**Yes (*n*, %)**
For 28 days	388 (67.1)
For 40 days	80 (13.8)
For 6 months	49 (8.4)
For life	61 (10.5)

**Table 3:** Respondents’ attitude about PEP for HIV.

Statement	Strongly Agree/ Agree (*n*, %)	Neutral (*n*, %)	Strongly Disagree/ Disagree (*n*, %)
Do you believe that training about PEP is important for a behavioral change in health care professionals?	435 (75.2)	80 (13.8)	63 (10.8)
Do you think there should be PEP guidelines present in working areas?	511 (88.4)	56 (9.6)	11 (1.9)
Do you think PEP declines the likelihood of being HIV-positive?	502 (86.8)	32 (5.5)	44 (7.6)
Do you think PEP is important if the exposure is not with blood of a known HIV-positive patient?	448 (77.5)	71 (12.2)	59 (10.2)
Do you believe HIV PEP prevents other infections (Hepatitis B and C)?	384 (66.4)	99 (17.1)	95 (16.4)

**Table 4:** Respondents’ practice of PEP after HIV occupational exposure in their professional lifetime.

Respondents’ Practice of PEP after Occupational Exposure	Responses (*n*, %)
Have you ever been in a risky situation?	
*Yes*	407 (70.4)
*No*	98 (16.9)
*Do not know*	73 (12.6)
Types of exposures	
*Blood splash*	214 (37.0)
*Needlestick injuries*	180 (31.1)
*Mucous splash*	96 (16.6)
*Others*	88 (15.2)
The time frame of occupational exposure	
*Within 3 months*	108 (18.6)
*Within 6 months*	132 (22.8)
*In the past one year*	211 (36.5)
*Do not remember the exact time frame*	127 (21.9)
Took PEP after exposure	
*Yes*	380 (65.7)
*No*	198 (34.2)
The reason the respondent took PEP	
*Exposure to blood from known HIV-positive patient*	125 (32.8)
*Exposure to an individual’s blood whose HIV status was unidentified*	109 (28.6)
*Injury from some sharp articles*	92 (24.2)
*Interaction with patient body fluids*	54 (14.2)
The time to start taking the PEP	
*Within an hour*	187 (49.2)
*After 2–6 h of exposure*	74 (19.4)
*After 6–10 h of exposure*	92 (24.2)
*After 72 h*	27 (7.1)
The length of time the responder took PEP for	
*3 days*	35 (6.0)
*15 days*	129 (22.3)
*28 days*	216 (56.8)
Reason for discontinuing the drug	
*Fear of adverse consequences*	201 (34.7)
*The amount of medicine used was adequate*	68 (11.7)
*Medicine was not effective*	238 (41.1)
*Other*	71 (12.2)

**Table 5:** Factors influencing occupational exposure reporting.

Variables	Pearson Chi-Squared Value	*p*-Value	Confidence Interval (CI)
Gender	0.72	0.531	0.24–1.31
Organization	0.35	0.5	0.32–1.64
Job category	7.64	0.0001	0.16–0.62
Experience	6.31	0.004	0.14–0.72
Age	0.89	0.406	0.27–1.24
Frequency of exposure	5.83	0.003	0.42–11.31
Knowledge on PEP	0.56	0.34	0.2–1.03

**Table 6:** Respondents’ opinion and attitude about the management of HIV occupational exposure in the healthcare institution they worked in.

Management of Occupational Exposure in the Healthcare Institution They Worked in	Yes (*n*, %)	No (*n*, %)	I Do Not Know (*n*, %)
Institute had a policy in black and white	311 (53.8)	89 (15.4)	178 (30.8)
Institute provided appropriate training to all employees	352 (60.8)	173 (30)	53 (9.2)
Institute established HIV occupational exposure reporting systems	218 (37.7)	227 (39.2)	133 (23.1)
Healthcare facility (HCF) had workers who could manage exposure and were accessible at all times	204 (35.4)	187 (32.3)	187 (32.3)
HCF established laboratory capacity for HIV testing	316 (54.6)	129 (22.3)	133 (23.1)
HCF created a protocol for the selection and administration of PEP antiretroviral regimens for HIV exposure.	176 (30.4)	158 (27.3)	244 (42.3)
Do you believe that an HCP who has been exposed to HIV should be tested?	458 (79.2)	31 (5.4)	89 (15.4)
Is HCF able to access resources with expertise in the selection and use of PEP?	191 (33.0)	107 (18.5)	280 (48.4)
Should HCF provide medication adherence counseling to assist HCPs in completing HIV PEP as required?	303 (52.4)	71 (12.3)	204 (35.3)
Should HCF provide counseling to HCP who may require aid in dealing with the emotional effects of exposure?	284 (49.2)	93 (16.1)	201 (34.7)
Is the HCP using antiretroviral PEP being followed for adverse effects of PEP by baseline and testing (every 2 weeks) and clinically evaluated?	235 (40.7)	53 (9.2)	290 (50.1)
Is there a protocol in place at your institute to encourage exposed HCPs to get follow-up testing?	170 (29.3)	155 (26.9)	253 (43.8)

## Data Availability

All data generated during the study are presented in this paper.
